# The Classification of Metastatic Spine Cancer and Spinal Compression Fractures by Using CNN and SVM Techniques

**DOI:** 10.3390/bioengineering11121264

**Published:** 2024-12-13

**Authors:** Woosik Jeong, Chang-Heon Baek, Dong-Yeong Lee, Sang-Youn Song, Jae-Boem Na, Mohamad Soleh Hidayat, Geonwoo Kim, Dong-Hee Kim

**Affiliations:** 1 Department of Bio-Industrial Machinery Engineering, Gyeongsang National University, Jinju 52828, Republic of Korea; jws7600@naver.com (W.J.); hdytsoleh@gmail.com (M.S.H.); 2 Department of Orthopaedic Surgery, Institute of Medical Science, Gyeongsang National University College of Medicine and Gyeongsang National University Hospital, Jinju 52727, Republic of Korea; changhuen64@gmail.com; 3 Department of Orthopaedic Surgery, Jinju Barun Hosptial, Jinju 52727, Republic of Korea; whatttary@hanmail.net; 4 Department of Orthopedic Surgery, Chinjujeil Hospital, Jinju 52709, Republic of Korea; songsangyoun@naver.com; 5 Department of Radiology, Gyeongsang National University School of Medicine, Jinju 52828, Republic of Korea; jbna@gnu.ac.kr; 6 Institute of Agriculture and Life Sciences, Gyeongsang National University, Jinju 52828, Republic of Korea

**Keywords:** spine, compression fractures, CNNs, SVM, Otsu’s binarization algorithm, Canny edge algorithm

## Abstract

Metastatic spine cancer can cause pain and neurological issues, making it challenging to distinguish from spinal compression fractures using magnetic resonance imaging (MRI). To improve diagnostic accuracy, this study developed artificial intelligence (AI) models to differentiate between metastatic spine cancer and spinal compression fractures in MRI images. MRI data from Gyeongsang National University Hospital, collected from January 2019 to April 2022, were processed using Otsu’s binarization and Canny edge detection algorithms. Using these preprocessed datasets, convolutional neural network (CNN) and support vector machine (SVM) models were built. The T1-weighted image-based CNN model demonstrated high sensitivity (1.00) and accuracy (0.98) in identifying metastatic spine cancer, particularly with data processed by Otsu’s binarization and Canny edge detection, achieving exceptional performance in detecting cancerous cases. This approach highlights the potential of preprocessed MRI data for AI-assisted diagnosis, supporting clinical applications in distinguishing metastatic spine cancer from spinal compression fractures.

## 1. Introduction

Cancer generated when primary cancer spreads to the spine, known as metastatic spine cancer (MSC), has continued to increase in the number of patients. To diagnose MSC, imaging techniques such as magnetic resonance imaging (MRI) and computed tomography (CT) scanning are used, and with simple radiography, the destruction of at least 30–50% of the vertebral body is necessary for MSC [1,2]. Accordingly, if a patient is misdiagnosed with a false negative reaction before destruction occurs so that treatment is delayed, tumors can exert pressure on the spinal cord and lead to serious complications such as neurological damage and diplegia, thus making rapid early detection important [3,4,5]. In addition, in order to detect MSC precisely, professions in fields such as radiology and oncology are necessary; however, it is difficult for all medical institutions to secure professionals. Consequently, to resolve these problems, the development of programs assisting cancer detection through the use of artificial intelligence (AI) is in high demand.

Constituting a common disorder where the incidence rate increases with a person’s age, spinal compression fractures are similar to cancer in MRI images, so that for accurate diagnosis, additional diagnoses such as osteoporosis tests are necessary. Nevertheless, the misdiagnosis rate amounts to 17% [6,7]. To overcome this, the necessity of a model that uses AI to distinguish between spinal compression fractures and MSC in diagnosis has emerged; however, related research has been insufficient. Consequently, the purpose of this study was to develop AI models that distinguish between spinal compression fractures and meta.

To achieve this, convolutional neural networks (CNN), which are widely used in the medical field today and exhibit high accuracy in detecting and classifying cancers such as lung cancer, breast cancer, and brain cancer, were utilized [8,9,10]. Support vector machine (SVM) techniques were also selected, which are well-suited for complex and high-dimensional data such as MRI data and exhibit notably high classification performance in AI-based cancer detection. Similar to CNNs, SVMs have been successfully applied in studies involving diverse types of cancer [11,12,13], including breast cancer, lung cancer, and skin cancer [14,15,16,17]. Otsu’s binarization algorithm and Canny edge detection algorithm processing methods were also used. By comparing these techniques and models, models capable of distinguishing between the characteristics of compression fractures and those of meta with high accuracy can be selected.

## 2. Materials and Methods

### 2.1. Patients

The ethical approval for this study was obtained from the Institutional Review Board (IRB). Data from 503 patients who underwent thoracic MRI examinations at Gyeongsang National University Hospital between 1 January 2019 and 30 April 2022 were collected. Among them, 255 patients with metallic implants due to fusion operation, which caused noise in MRI images, were excluded. As a result, images from 248 patients were analyzed.

Metastasis was defined as cases diagnosed as spinal metastasis through radiographic examination (MRI) and histopathology. Primary spinal tumors, such as schwannomas, spinal meningiomas, osteochondromas, as well as conditions like spinal tuberculosis, were excluded. Patients without metastasis were classified as non-metastasis (NSC). All non-metastasis cases with compression fractures were identified and classified based on radiologic examination findings.

Finally, data on 135 MSC patients and 113 NSC patients were acquired. Among the 113 NSC patients, 43 were diagnosed with compression fractures.

Among the 248 patients included in the analysis, 138 (55.6%) were male and 110 (44.4%) were female, with a male-to-female ratio of 1.25:1. Among MSC patients, 79 (56.3%) were male and 56 (43.7%) were female, while in the NSC patient group, 62 (54.9%) were male and 51 (45.1%) were female. The total mean age was 64.48 ± 15.5 years, with the mean age for MSC patients being 64.3 ± 13.7 years and for NSC patients being 64.7 ± 17.5 years. The largest proportion of patients fell within the 70–79 age range (n = 76, 30.6%). The majority of MSC patients were in the age groups of 60–69 years (n = 40, 29.6%) and 70–79 years (n = 40, 29.6%).

Primary tumors diagnosed through radiologic examination and histopathology included those originating from the lung, genitourinary system, hepatobiliary system, breast, colorectal region, hematopoietic system, thyroid, stomach, skin, gynecologic system, and tumors of unknown origin.

### 2.2. Image Acquisition and Data Structure

The spine images were obtained exclusively from Gyeongsang National University Hospital using a 3.0 Tesla MRI scanner (Ingenia Elition; Philips Healthcare, Orlando, FL, USA). The MRI images for each patient were acquired using the shortest possible echo time (TE), repetition time (TR), and slice thickness settings.

MRI images provided by the Department of Radiology consisted of MSC data and non-meta data, which were sagittal view images. In addition, each image consisted of T1-weighted images, T2-weighted images, and contrast-enhanced T1-weighted images. In order to distinguish between meta data and non-meta data, before constructing the datasets, data on MSC and compression fractures were grouped by a radiologist. The final dataset consisted of 362 MSC images, 262 MSC compression fracture (MSCF) images, 345 NSC images, and 207 NSC compression fracture (NSCF) images using sagittal view images measuring 448 × 448.

To obtain the characteristics of meta, non-meta, and non-meta compression fracture images from specific datasets and compare the classification performance, acquired MRI images were divided into 4 datasets (dataset 1 to 4) as shown in Table 1. In Table 1, class 1, 2, 3, and 4 are MSC images (MSCI), MSCF images (MSCFI), NSC images (NSCI), and NSCF images (NSCFI).

### 2.3. Preprocessing Methods

**Figure 1:**
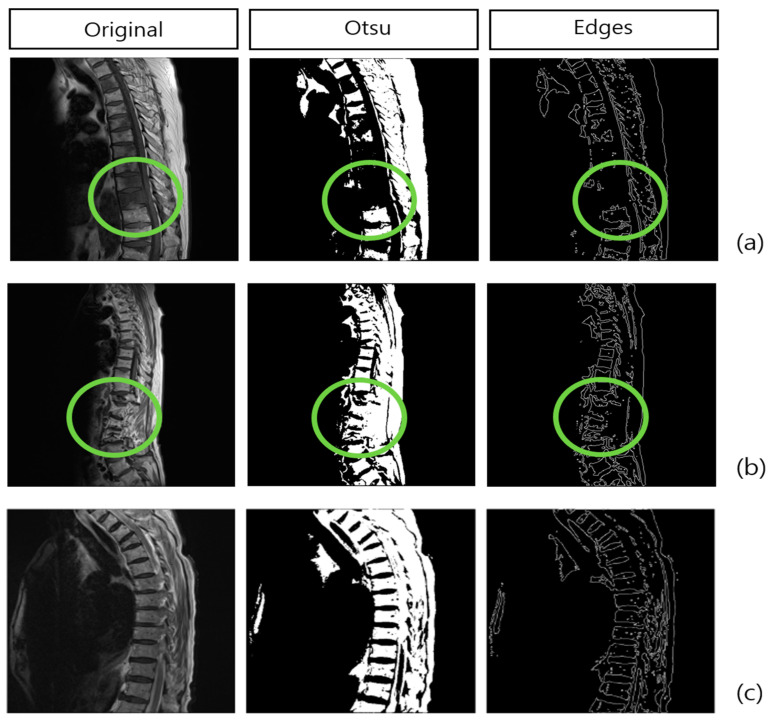
Structural changes revealed when Otsu’s binarization algorithm and Canny edge detection algorithm preprocessing methods were used in: (**a**) MSC case; (**b**) NSCF case; and (**c**) NSC case. The green circles are utilized as key criteria for diagnosing diseases in each case.

As shown in Figure 1, Otsu’s binarization algorithm automatically finds the threshold values capable of best separating an image’s two classes (background and foreground) and is used to separate objects or to extract edges from images. This algorithm has been used in research on AI models assisting breast cancer diagnosis [18,19,20]. Here, the mean accuracy of cancer detection was 98.47%, thus exhibiting high accuracy [21]. The MRI images used for meta detection were also grayscale images; Otsu’s binarization algorithm preprocessing technique was applied. Because metadata appear black in T1-weighted images and white in contrast-enhanced T1-weighted images, unlike the original spinal structure appearing in NSC cases, the disarranged structure was used. The principle of this algorithm is as follows. With the threshold value as the standard, the two classes were separated, defining class 1 as pixels below the threshold value and class 2 as pixels equal to or above the threshold value. Subsequently, the pixel ratio and the mean grayscale value were calculated for each class. Then, with a mathematical expression for maximizing the variance between the two classes, the variance between the two classes, defined as the difference between the mean value of each class and the overall mean value, was calculated, and the threshold value where the variance was maximal was selected. As for detailed information on Otsu’s binarization algorithm, see reference [18].

As in Figure 1, the Canny edge detection algorithm identifies and extracts objects’ boundaries and edges from images. Moreover, the Canny edge detection algorithm is one of the most widely used boundary detection techniques in the field of image processing [22]. This algorithm provides an optimal approach for boundary detection and is very effective for distinguishing between objects and backgrounds within images. In particular, it makes robust boundary detection possible, even in images that include noise [23]. With MRI images, there exist both noises generated by devices’ sensors or electronic mechanisms and noise generated inside tissues due to complex reflection patterns. Since the medical imaging field demands high accuracy, the diverse methods of eliminating noise and performing boundary detection have been analyzed [24,25,26] and were also applied to this study. The main process of the Canny edge detection algorithm is as follows. First, a Gaussian filter is used to eliminate noise from the images, and a Sobel filter is used to calculate the gradient in each pixel to find out where changes in the images’ brightness are the greatest. The boundary strength of each pixel in the images in the horizontal and vertical directions is obtained in the stage above, which determines the boundary direction and size of each pixel. Next, for accurate boundary detection, only the maximum values according to the gradient direction remain, and the rest is eliminated. Through this, erroneous boundary detection that can be generated in non-boundary domains is suppressed. Next, the threshold value is determined according to the gradient strength, and boundaries are distinguished into strong and weak boundaries. Finally, individual boundaries are connected, thus making accurate boundary detection possible. As for detailed information on the Canny edge detection algorithm, refer to reference [23].

As mentioned above, Otsu’s binarization algorithm and the Canny edge detection algorithm allow for lighter training compared to grayscale MRI images divided into 256 levels. Additionally, they are expected to enhance noise suppression, reduce execution time, and improve accuracy in cancer screening models.

### 2.4. Model Development

The composition of each layer in the CNN model used in this study was as follows. Thirty-two filters were used in the first convolution layer, each of which measured 3 × 3, and the rectified linear unit (ReLU) was used as the activation function. A max pooling layer measuring 2 × 2 was then applied to reduce the sizes of the feature maps.

In the second convolutional layer, 64 filters were applied, and a filter identically measuring 3 × 3 and the ReLU activation function were used. Then, after passing through the second max pooling layer, the characteristics were extracted, and the sizes were reduced. In the third convolutional layer, 128 filters were used to extract even more complex characteristics, and the max pooling layer was applied. Finally, the flattened layer was used for transformation into a one-dimensional vector, and dense layers with 128 neurons were added to learn the final characteristics. The sigmoid activation function was used on the final output layer in the binary classification, and the softmax activation function was used in the multiclass classification.

With the SVM model, data were classified by using linear kernels (kernels = “linear”). Linear kernels are appropriate for cases where data are linearly distinguishable and make prompt and efficient learning possible without increasing a model’s complexity. Also, a regularization parameter (C = 1.0) was used to prevent overfitting. In addition, there are cases where the SVM algorithm exhibits high accuracy when preprocessed with Otsu’s binarization algorithm [27,28,29], and this, along with CNNs, is diversely used in medical fields including the detection of cancers such as lung cancer, breast cancer, and cervical cancer [27,28,30].

### 2.5. Performance Evaluation Criteria for the Models Developed

The scores in the results were classified into six categories. Sensitivity represents the ratio of data that a model accurately predicts as actually belonging to a specific class. Specificity represents the ratio of data that a model accurately predicts as actually not belonging to a specific class. A positive predictive value (PPV) represents the ratio of data actually belonging to a specific class as predicted by the model, or the accuracy of such predictions. A negative predictive value (NPP) represents the ratio of data actually not belonging to a specific class as predicted by the model, or the accuracy of such predictions. With a higher F1-score value, which is a harmonic mean representing a balance between precision and recall, a model’s classification performance is improved. Accuracy represents the ratio of a model’s accurate predictions out of the total data.

## 3. Results

The results of this study were determined on the basis of the results of each model in dataset 3 and dataset 4 in the dataset classification shown in Table 1. As for dataset 1 and dataset 2, the models’ mean accuracy scores amounted to 0.54 and 0.57, respectively, and were excluded from the results because they were lower than the accuracy in dataset 3 and dataset 4 (0.83–0.98). Between dataset 3 and dataset 4, the models were able to classify cancer more accurately.

### 3.1. Patient Characteristics

A total of 248 patients (aged 25–97) were included. Out of 135 MSC patients and 113 NSC patients, NSC patients included 43 NSCF patients.

### 3.2. Models Using Dataset 3 with the Exclusion of Spinal Compression Fractures

Table 2 shows the results of models conducted with the dataset 3 with the exclusion of spinal compression fractures. When each preprocessing method is implemented on the MRI data given in this table, the effects on the performance of models using the CNN and SVM techniques can be evaluated and compared.

With T1-weighted images, data trained using original MRI images in the SVM model exhibited the highest accuracy (0.986). With T2-weighted images and contrast-enhanced T1-weighted images, the model using original MRI images in the CNN model exhibited the highest accuracy (0.986 and 0.987, respectively). The results of models with high sensitivity were as follows. With T1-weighted images, when the SVM model was trained with data preprocessed with Otsu’s binarization algorithm and the Canny edge detection algorithm, the highest score was obtained (1.00). With T2-weighted images, both the CNN model using original MRI data and the SVM model using data preprocessed with the Canny edge detection algorithm yielded the highest scores (both 1.00). With contrast-enhanced T1-weighted images, both the CNN model using data preprocessed with Otsu’s binarization algorithm and the SVM model using data preprocessed with the Canny edge detection algorithm yielded the highest scores (0.984 and 0.990, respectively). The SVM model using data preprocessed with the Canny edge detection algorithm scored high on sensitivity.

### 3.3. Models Using Dataset 4 with the Inclusion of Spinal Compression Fractures

Table 3 shows the results of models conducted with dataset 4 including NSCF. With T1-weighted images, the CNN and SVM models both exhibited the highest performance (0.982 and 0.971, respectively) when original MRI images were used. In particular, the CNN model yielded high scores on both sensitivity and accuracy regarding MSC data (1.000 and 0.982, respectively). With T2-weighted images, the SVM model, which used original MRI images, exhibited high sensitivity and accuracy (1.000 and 0.982, respectively). With contrast-enhanced T1-weighted images, the CNN model, which used Otsu’s binarization algorithm as preprocessing data, exhibited high sensitivity and accuracy (1.000 and 0.954, respectively).

## 4. Discussion

In this study, the developed CNN model, which used MRI datasets, exhibited the most outstanding performance. However, it is more dangerous to misdiagnose MSC patients as NSC patients than to perceive NSC patients as MSC patients. Consequently, sensitivity, or scores on whether or not MSC cases were detected as MSC cases, was established as an essential criterion. As for sensitivity scores, which were taken into consideration to detect MSC cases well, there were cases where models developed after undergoing preprocessing methods using Otsu’s binarization algorithm and the Canny edge detection algorithm exhibited better performance.

In particular, as for dataset 3, with T1-weighted images, the SVM model, which was trained after being preprocessed with Otsu’s binarization algorithm, yielded the highest sensitivity (1.000) and accuracy (0.980) scores. Likewise, with T2-weighted images and contrast-enhanced T1-weighted images, when the model was preprocessed by using Otsu’s binarization algorithm and the Canny edge detection algorithm, there were cases exhibiting low accuracy (0.928–0.979) but high sensitivity scores (0.980–1.000). Consequently, it was successfully confirmed that when a model was trained after preprocessing using Otsu’s binarization algorithm and the Canny edge detection algorithm, it could accurately judge MSC cases, thus decreasing the misdiagnosis rate.

When sensitivity and accuracy were comprehensively considered, the best models for MSC diagnosis assistance were as follows: with T1-weighted images, where accuracy was high, the SVM model was best (accuracy: 0.986); and, with T2-weighted images and contrast-enhanced T1-weighted images, the CNN model was best (accuracy: 0.986, 0.987). In addition, for predictions with new MRI images, these models took less than one second. Consequently, with prompt and high accuracy, they are expected to assist MSC well.

Because the high-performance model developed earlier (dataset 3) was trained with the dataset created with the exclusion of NSCF, there were limitations in distinguishing between compression fractures and meta. Consequently, a model capable of classifying even NSCF was selected in dataset 4. As for dataset 4, where the model was created by adding NSCF to the dataset, the case where the model was trained with a CNN through the use of T1-weighted images and MRI images was selected as the perfect one. In this case, the overall accuracy was 0.982, thus exhibiting a high level. In particular, as for MSC cases, the model made judgments with an accuracy on the 1.00 level (sensitivity, etc.) and likewise yielded sensitivity and PPV scores of 0.875 and 0.933, respectively, on NSCF.

While some models did exhibit high performance, some revealed limitations in that when sensitivity scores were high for MSC cases, those for NSCF were low. One representative case is that of the Canny edge detection algorithm on T1-weighted images in Table 3. Whereas the sensitivity score on meta cases was 1.000, thus predicting actual meta cases as meta cases well, the PPV score was 0.782, thus being low, indicating that data predicted as meta included many other non-meta images. Here, the score on sensitivity to NSCF was 0.600, thus being low, which shows a limitation in that the model failed to properly predict NSCF. However, the PPV score was 1.000, which means that the prediction of NSCF was based on a good grasp and classification of the characteristics of NSCF. In addition, most cases exhibited an accuracy of 0.75 or above. Consequently, as for dataset 4, when a model was created by using original MRI images and data that had undergone Otsu’s algorithm preprocessing process, the possibility of finding the characteristics solely of NSCF was confirmed, despite limitations in accuracy, so that the model could not be seen as perfect.

In a case similar to this study, previous research has studied a model that detects compression fractures with high sensitivity (95.7%) at a low false-positive rate, thus confirming the existence of characteristics solely of NMSC [31,32] essential criteria. The quantity of data will be increased, and methods including the implementation of segmentation and the application of diverse models will be used. Compared to previous large-scale retrospective studies on spinal metastasis, the dataset in this study showed similarities in gender ratio, mean age, and the age group with the highest patient distribution [33]. By diversifying the data collection institutions, the representativeness of the dataset for a broader population could be further enhanced.

In this study, we aimed to explore the initial potential of the model as a broader diagnostic tool to assist, rather than replace, clinical expertise in their diagnoses. With future improvements to the model, meaningful comparisons between standalone clinical diagnosis and model-assisted diagnosis are anticipated to become feasible. Consequently, in the future, it will be possible to construct high-performance spinal cancer diagnosis models that can accurately detect the characteristics of NSCF solely based on MRI images.

This study had a limitation in that the dataset was acquired from a single institution. As mentioned above, the more similar the characteristics of the collected dataset are to the broader population, the greater its representativeness and generalizability will be. Therefore, it will be necessary to collect datasets from more diverse sources in the future to enhance their similarity to the general population.

Additionally, patients with implants were excluded from the dataset in this study. To analyze a broader range of patient groups in the future, improvements to the model will be required. This will be a critical challenge in determining the model’s generalizability.

## 5. Conclusions

To develop AI models distinguishing between MSC and NSCF, this study used CNN and SVM techniques and applied image preprocessing methods using Otsu’s binarization algorithm and the Canny edge detection algorithm. As a result, both the T1W1-based CNN model and the SVM model recorded high accuracy (0.982 and 0.971, respectively) and sensitivity (1.000 and 0.978, respectively), and preprocessing methods using Otsu’s binarization algorithm and the Canny edge detection algorithm were confirmed to contribute to performance improvement.

In addition, the models developed in this study exhibited especially outstanding performance in sensitivity to MSC (1.00), thus confirming their possibility of being used as diagnostic assistance systems aiding prompt and accurate diagnosis. However, because this study was conducted with a focus on limited datasets and specific MRI images, additional research using more diverse images is necessary in the future.

In future research, the goal will be to construct a more precise diagnostic model by increasing the quantity of data and applying diverse image preprocessing techniques and classification models. Through this, it will become possible, in clinical practice, to utilize a functional diagnostic assistance system capable of distinguishing between MSC and NSCF more effectively.

## Figures and Tables

**Table 1:** The MRI dataset used in this study; MSCI, MSCCFI, NMSCI, and NMSCCFI.

Dataset	Class 1	Class 2	Class 3	Class 4
1	MSCI	MSCFI	NSCI	NSCFI
2	MSCI and MSCFI		NSCI and NSCFI	
3	MSCI and MSCFI		NSCI	-
4	MSCI and MSCFI		NSCI	NSCFI

**Table 2:** The MRI datasets used in this study; MSCI, MSCCFI, NMSCI, and NMSCCFI.

Dataset 3: Meta/Non-Meta			Sensitivity	Specificity	PPV	NPV	F1-Score	Accuracy
MRI	Model	Preprocessing						
Sagittal _t1	CNN	Standard	0.943	0.989	0.980	0.968	0.962	0.973
		Otsu	0.940	0.990	0.980	0.969	0.959	0.973
		Edge	0.940	0.979	0.959	0.969	0.950	0.966
	SVM	Standard	0.986	0.980	0.989	0.980	0.990	0.986
		Otsu	1.000	0.938	0.970	1.000	0.985	0.980
		Edge	1.000	0.814	0.928	1.000	0.963	0.945
Sagittal _t2	CNN	Standard	1.000	0.978	0.964	1.000	0.982	0.986
		Otsu	0.918	1.000	1.000	0.960	0.957	0.972
		Edge	0.915	0.959	0.915	0.959	0.915	0.945
	SVM	Standard	0.980	0.978	0.990	0.957	0.985	0.979
		Otsu	0.990	0.959	0.979	0.979	0.985	0.979
		Edge	1.000	0.784	0.931	1.000	0.964	0.945
Sagittal Contrast-enhanced _t1	CNN	Standard	0.979	0.990	0.979	0.990	0.979	0.987
		Otsu	0.984	0.978	0.969	0.989	0.976	0.980
		Edge	0.933	0.957	0.933	0.957	0.933	0.947
	SVM	Standard	0.978	0.983	0.989	0.967	0.984	0.980
		Otsu	0.980	0.943	0.970	0.962	0.975	0.967
		Edge	0.990	0.811	0.907	0.977	0.947	0.928

**Table 3:** The highest evaluation scores for each preprocessing method and training technique performed on the basis of dataset 4 including NSCF.

Dataset 4: Meta/Non-Meta/Non-Meta with Compression Fracture			Metrics for Meta					Metrics for Non-Meta					Metrics for Non-Meta Compression Fracture					Accuracy
MRI	Model	Preprocessing	Sensitivity	Specificity	PPV	NPV	F1-Score	Sensitivity	Specificity	PPV	NPV	F1-Score	Sensitivity	Specificity	PPV	NPV	F1-Score	
Sagittal _t1	CNN	Standard	1.000	1.000	1.000	1.000	1.000	0.984	0.982	0.968	0.991	0.9765	0.875	0.994	0.933	0.987	0.903	0.982
		Otsu	0.980	0.958	0.969	0.972	0.975	0.978	0.976	0.936	0.992	0.957	0.889	1.000	1.000	0.980	0.941	0.965
		Edge	0.978	0.949	0.957	0.974	0.967	0.938	0.975	0.938	0.975	0.938	0.871	0.956	0.931	0.972	0.900	0.947
	SVM	Standard	0.978	0.987	0.989	0.975	0.984	1.000	0.968	0.922	1.000	0.960	0.903	1.000	1.000	0.979	0.949	0.971
		Otsu	0.989	0.866	0.888	0.986	0.936	0.923	0.992	0.980	0.967	0.951	0.767	1.000	1.000	0.952	0.868	0.929
		Edge	1.000	0.630	0.782	1.000	0.878	0.621	0.991	0.973	0.835	0.758	0.600	1.000	1.000	0.963	0.750	0.835
Sagittal _t2	CNN	Standard	0.952	0.953	0.952	0.954	0.952	0.946	0.973	0.946	0.974	0.946	0.936	0.986	0.936	0.986	0.936	0.947
		Otsu	0.988	0.904	0.914	0.987	0.950	0.831	0.991	0.980	0.916	0.899	1.000	0.986	0.923	1.000	0.960	0.935
		Edge	0.960	0.971	0.980	0.944	0.970	0.966	0.955	0.966	0.955	0.918	0.982	0.941	0.909	1.000	1.000	0.959
	SVM	Standard	1.000	0.969	0.981	1.000	0.991	0.976	0.992	0.976	0.992	0.976	0.913	1.000	1.000	0.987	0.955	0.982
		Otsu	0.980	0.914	0.942	0.970	0.960	0.913	0.984	0.955	0.968	0.933	0.875	0.993	0.955	0.980	0.913	0.947
		Edge	0.990	0.685	0.805	0.980	0.888	0.698	0.983	0.949	0.877	0.804	0.600	1.000	1.000	0.949	0.750	0.852
Sagittal Contrast-enhanced_t1	CNN	Standard	0.966	0.906	0.914	0.963	0.939	0.912	0.966	0.929	0.957	0.920	0.786	0.986	0.917	0.960	0.846	0.919
		Otsu	1.000	0.939	0.948	1.000	0.973	0.922	1.000	1.000	0.956	0.959	0.833	0.981	0.833	0.981	0.833	0.954
		Edge	0.990	0.916	0.944	0.985	0.967	0.923	0.984	0.960	0.968	0.941	0.842	1.000	1.000	0.981	0.914	0.954
	SVM	Standard	0.966	0.965	0.966	0.965	0.966	0.982	0.966	0.931	0.991	0.956	0.900	1.000	1.000	0.980	0.947	0.960
		Otsu	0.989	0.913	0.929	0.987	0.958	0.902	0.982	0.965	0.948	0.932	0.790	0.987	0.882	0.974	0.833	0.936
		Edge	0.947	0.769	0.833	0.923	0.887	0.794	0.909	0.833	0.885	0.813	0.333	1.000	1.000	0.941	0.500	0.838

## Data Availability

The data presented in this study are available on request from the corresponding author due to privacy.
